# Assessment of iPS Cell-derived Dopaminergic Progenitor Cells Properties with Long-term Passaging and Amplification

**DOI:** 10.14789/jmj.JMJ22-0034-R

**Published:** 2023-01-26

**Authors:** TOSHIKI MINOBE, RISA NONAKA, TAKAHIRO SHIGA, KEI-ICHI ISHIKAWA, NOBUTAKA HATTORI, WADO AKAMATSU

**Affiliations:** 1 Center for Genomic and Regenerative Medicine, Juntendo University Graduate School of Medicine, Tokyo, Japan; 1 Center for Genomic and Regenerative Medicine, Juntendo University Graduate School of Medicine, Tokyo, Japan; 2 Department of Clinical Data of Parkinson's Disease, Juntendo University Graduate School of Medicine, Tokyo, Japan; 2 Department of Clinical Data of Parkinson's Disease, Juntendo University Graduate School of Medicine, Tokyo, Japan; 3 Department of Neurology, Juntendo University School of Medicine, Tokyo, Japan; 3 Department of Neurology, Juntendo University School of Medicine, Tokyo, Japan; 4 Department of Research and Development for Organoids, Juntendo University School of Medicine, Tokyo, Japan; 4 Department of Research and Development for Organoids, Juntendo University School of Medicine, Tokyo, Japan; 5 Neurodegenerative Disorders Collaborative Laboratory, RIKEN Center for Brain Science, Saitama, Japan; 5 Neurodegenerative Disorders Collaborative Laboratory, RIKEN Center for Brain Science, Saitama, Japan

**Keywords:** induced pluripotent stem cell, neural stem cell, neurosphere, genomic stability, dopaminergic neuron

Dopaminergic (DA) progenitor cells derived from iPSCs have the potential as a resource of regenerative medicine for Parkinson's disease. Since the safety of cells derived from iPSCs requires a lot of quality assurance, mainly safety verification, autologous transplantation is difficult for practical use, and it is necessary to prepare cell stocks with guaranteed safety. Since undifferentiated iPSCs have genomic instability^1)^, there is a risk of causing genomic structural abnormalities when large numbers are cultured for creating large stocks. On the other hand, differentiated cells are presumed to have a relatively stable genome structure, so we thought it would be safer to amplify them after induction. Neurospheres are an efficient method to expand the number of neural stem cells by passaging. However, it was unclear how long these properties, such as regional identity, proliferation, neuronal differentiation, and genomic stability, would be maintained. To reveal this problem, we passaged the neural stem cells using neurospheres differentiated from two types of healthy control-iPS cells from different somatic cells (201B7 and aTKA4) to ensure versatility and tried to evaluate the changes in their properties.

Primary Neurospheres (PNS) were induced from healthy control-derived human iPSCs (201B7 and aTKA4) after treatment with three inhibitors as previously described with purmorphamine and CHIR99021 to provide the regional identity of the ventral midbrain^2)^. Neurospheres were passaged every seven days to form secondary neurospheres (2NS), tertiary neurospheres (3NS), and so on up to 10NS. Then, the properties of neurospheres were evaluated by cell proliferation, qPCR, and Karyotype analysis. Then, neurospheres were differentiated into DA neurons in vitro and evaluated differentiation efficiency by immunostaining against Tyrosine Hydroxylase (TH), a dopaminergic neuron marker, and β-III-tubulin, a neuron marker.

The growth rates of neurospheres calculated by cell counts showed that cell proliferation of 201B7 and aTKA4 were not changed until 10NS. qPCR analysis revealed that the expression levels of midbrain markers (EN1, FOXA2, LMX1A, and TH) were not changed after 2NS to 10NS, and the expression levels of forebrain markers (SIX3 and FOXG1) and hindbrain markers (HOXB4 and HOXC4) were low at 2NS and 3NS. Similar results were obtained for 201B7 and aTKA4. Immunocytochemical staining was performed with antibodies against TH. An immunofluorescence study performed on 2NS and 10NS showed that the percentage of TH+ neurons was 34.7±7.2% (201B7-2NS), 29.0± 3.2% (201B7-10NS), 27.9±6.7% (aTKA4-2NS), 24.1±4.0% (aTKA4-10NS). These results showed that dopamine neuronal differentiation efficiency is stable from 2NS to 10NS. Chromosomal integrity, karyotyping, and genotyping of neurospheres were analyzed by Karyostat Array (ThermoFisher SCIENTIFIC). Whole Genome View showed that no new significant mutations were detected in 201B7 and aTKA4 after nine passages. Undifferentiated iPSC genomes are known to be unstable, and they are easily mutated by passaging. However, karyotyping results showed that they rarely mutate after their differentiation into neurospheres and subsequently passaging.

Ventral midbrain-specific neurospheres induced by our method can be maintained with stable quality for at least 70 days in any iPS cell lines. These results suggest that given the stability of the genome, neurosphere-based passaging and amplification is the preferred method for large-scale culture of neural stem cells.

## Funding

This work was funded by MEXT-Supported Programs for the Strategic Research Foundation at Private Universities (S1411007) and the Practical Research Project for Rare/Intractable Diseases (JP20ek0109429 to K.I., N.H., and W.A.) from AMED.

## Author contributions

NR, TS, KI, and WA conceived and designed the experiments. TM and NR performed the experiments and analyzed the data. TM, KI, NH and WA wrote and revised the manuscript. All authors have reviewed and approved the manuscript.

## Conflicts of interest statement

The authors declare that there are no conflicts of interest.
